# Youth's Awareness of and Reactions to *The Real Cost* National Tobacco Public Education Campaign

**DOI:** 10.1371/journal.pone.0144827

**Published:** 2015-12-17

**Authors:** Jennifer C. Duke, Tesfa N. Alexander, Xiaoquan Zhao, Janine C. Delahanty, Jane A. Allen, Anna J. MacMonegle, Matthew C. Farrelly

**Affiliations:** 1 RTI International, Research Triangle Park, NC, United States of America; 2 Center for Tobacco Products, U.S. Food and Drug Administration, Silver Spring, MD, United States of America; 3 Department of Communication, George Mason University, Fairfax, VA, United States of America; Legacy, Schroeder Institute for Tobacco Research and Policy Studies, UNITED STATES

## Abstract

In 2014, the Food and Drug Administration (FDA) launched its first tobacco-focused public education campaign, *The Real Cost*, aimed at reducing tobacco use among 12- to 17-year-olds in the United States. This study describes *The Real Cost* message strategy, implementation, and initial evaluation findings. The campaign was designed to encourage youth who had never smoked but are susceptible to trying cigarettes (susceptible nonsmokers) and youth who have previously experimented with smoking (experimenters) to reassess what they know about the “costs” of tobacco use to their body and mind. *The Real Cost* aired on national television, online, radio, and other media channels, resulting in high awareness levels. Overall, 89.0% of U.S. youth were aware of at least one advertisement 6 to 8 months after campaign launch, and high levels of awareness were attained within the campaign’s two targeted audiences: susceptible nonsmokers (90.5%) and experimenters (94.6%). Most youth consider *The Real Cost* advertising to be effective, based on assessments of ad perceived effectiveness (mean = 4.0 on a scale from 1.0 to 5.0). High levels of awareness and positive ad reactions are requisite proximal indicators of health behavioral change. Additional research is being conducted to assess whether potential shifts in population-level cognitions and/or behaviors are attributable to this campaign. Current findings demonstrate that *The Real* Cost has attained high levels of ad awareness which is a critical first step in achieving positive changes in tobacco-related attitudes and behaviors. These data can also be used to inform ongoing message and media strategies for *The Real Cost* and other U.S. youth tobacco prevention campaigns.

## Introduction

Every day in the United States, more than 2,600 youth younger than age 18 smoke their first cigarette, and nearly 600 become daily smokers [1]. Although cigarette smoking rates among middle and high school-age youth have decreased markedly in recent years [2], an estimated 5.6 million Americans younger than age 18 at present are projected to die prematurely of smoking-related disease unless rates decline further [3]. Many more will live with a tobacco-related illness [3].

As a way to reduce the enormous public health burden of tobacco, the Family Smoking Prevention and Tobacco Control Act has given the Food and Drug Administration (FDA) the authority to take action to protect children, encourage smokers to quit, and reduce tobacco-related disease and death. The law also enables FDA to educate the public, especially young people, about the dangers of tobacco products. Research shows that public education mass media campaigns can be used to change attitudes and beliefs about tobacco use and reduce smoking prevalence [4–7]. In fact, the Centers for Disease Control and Prevention (CDC) considers mass media campaigns to be a “best practice” for tobacco control [8]. On February 11, 2014, FDA’s Center for Tobacco Products launched *The Real Cost*, the first of several complementary national public education campaigns designed to reduce tobacco use among U.S. youth aged 12 to 17. The initial phase of the campaign is designed to prevent smoking the initiation of cigarette smoking among youth who have never smoked but are susceptible to smoking (susceptible nonsmokers) and to discourage further smoking among youth who have experimented with smoking in the past (experimenters). The purpose of this paper is to (1) describe the rationale and implementation of *The Real Cost*; and (2) report results from the first follow-up evaluation of *The Real Cost*, with a focus on youth’s awareness of the campaign and their reactions to four advertisements that aired on television and the Internet prior to the first follow-up data collection.

### The Real Cost Campaign

#### Theoretical Basis for the Campaign Strategy

Systematic review of the literature suggests that campaigns with a solid theoretical basis are more likely to be effective [9]. Drawing from broad behavior change theories, such as the theory of reasoned action [10] and social cognitive theory [11], *The Real Cost* campaign seeks to influence tobacco use intentions and behavior among youth aged 12 to 17. The specific target population of the campaign includes nonsmokers who are susceptible to initiation as indicated by current intentions and cigarette experimenters who have not yet transitioned into established smoking [12,13]. According to the theory of reasoned action and social cognitive theory, intentions to abstain from smoking may be formed and reinforced by youth’s attitudinal and social normative beliefs about smoking and by their perceived ability to reject smoking in the future. The overall campaign strategy of *The Real Cost* is to influence these underlying beliefs through creative messaging.

#### Factors Expected to Influence Campaign Effectiveness

Among factors that may influence campaign effectiveness are audience exposure to and receptivity toward advertisements. Research suggests that insufficient exposure is a prominent reason that behavior change campaigns do not produce effects [14]. According to guidelines for best practices in tobacco control [8], campaign messages should reach at least 75% of the target audience, each quarter of the year. However, exposure to a media campaign does not guarantee that youth will engage with its messages in any meaningful way. The elaboration likelihood model [15] indicates that in order for a message to produce strong, lasting effects, it should be fully attended to by the viewer and the message content must be adequately processed and understood. Changes in tobacco-related beliefs and attitudes are, in part, a function of the level of cognitive processing or “elaboration” that occurs in response to campaign messages, and a wide body of research in the health communication literature has established measures of advertising effectiveness from this perspective. Tobacco-related media messages have been evaluated with scales of perceived effectiveness (PE) that capture appraisals and ratings of ad quality (e.g., Davis et al. [16,17]; Niederdeppe et al. [18]; Wong and Cappella [19]). The PE scale used in this study, in particular, has been validated as predictive of tobacco-related beliefs, attitudes, behavioral intentions, and smoking-related behaviors (Davis et al., 2015, unpublished work) [17].

If a campaign generates sufficient exposure and the audience considers campaign advertisements to be effective, it is estimated to take approximately 3 to 6 months to detect population-level campaign awareness and 12 to 18 months to produce behavior change [8]. To assess the impact of *The Real Cost*, FDA is conducting a systematic evaluation using a longitudinal design to monitor the campaign’s implementation and impact. The current study focuses on ad awareness and perceived ad effectiveness, two outcomes that are suitable for examination given the 6- to 8-month time frame between campaign launch and data collection.

#### Campaign Message Strategy and Implementation

**Fig 1:**
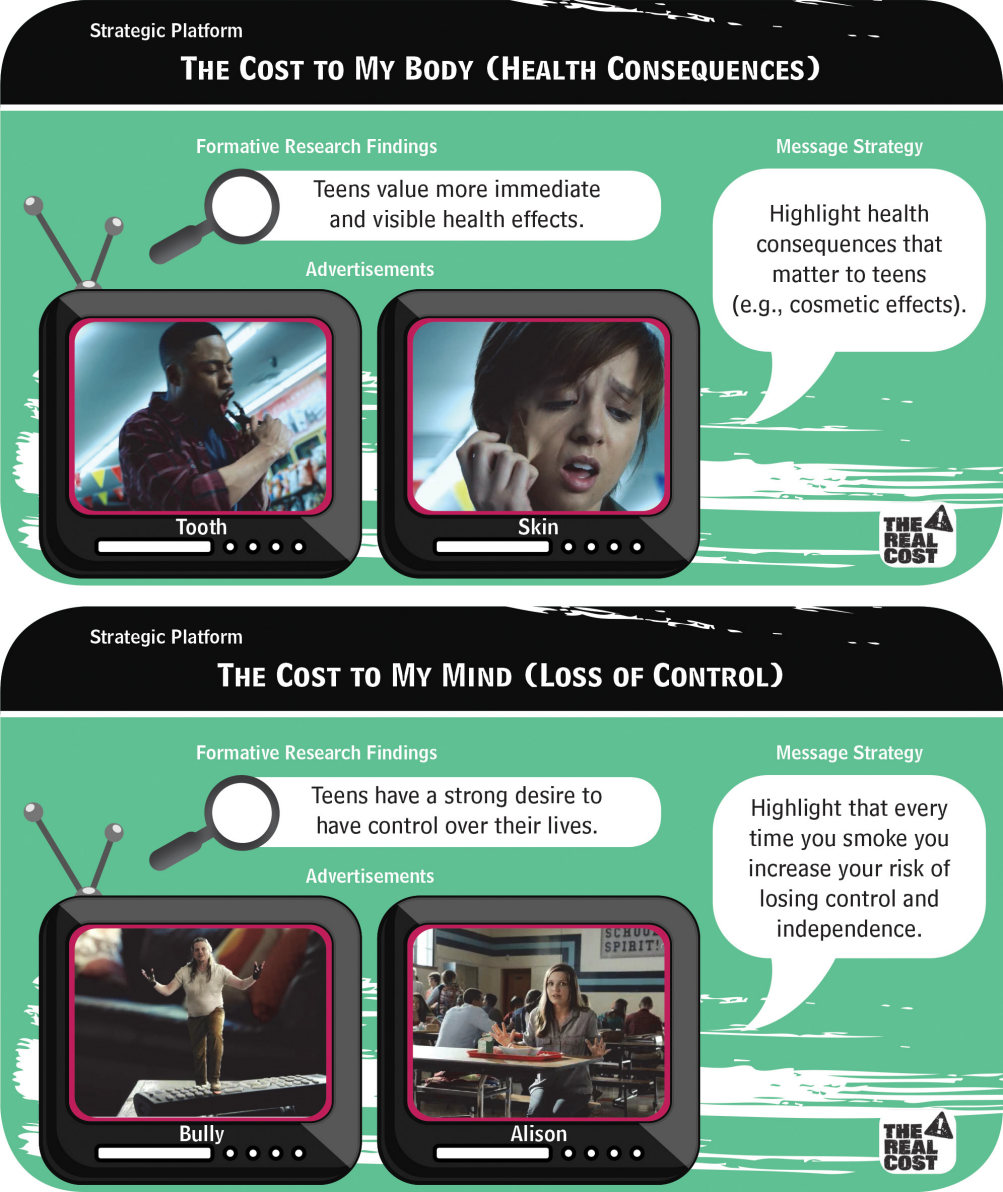
*The Real Cost* Message Concepts.

FDA launched *The Real Cost* in 75 media markets in February 2014. *The Real Cost’s* strategic message platform was informed by previous research [4,8] as well as findings showing that youth intention to smoke is correlated with beliefs about (1) the health consequences of tobacco use, (2) tobacco use leading to the loss of control and independence, and (3) the dangerous chemicals in each cigarette [20]. Based on these results, FDA worked with FCB Garfinkel advertising agency to conduct formative research to inform the development of the campaign brand and campaign advertisements. Formative research consisted of 12 focus groups with a total of more than 60 participants and quantitative online copy testing with over 1,600 respondents. Participants were aged 12 to 17 and either self-reported as nonsmokers who were susceptible to smoking in the future (i.e., susceptible nonsmokers) or as having previously experimented with smoking (i.e., experimenters). Informed from insights gained through formative research with the target audience, *The Real Cost* is designed to encourage teen susceptible nonsmokers and experimenters to reassess what they know about the “costs” of tobacco use to their body and their mind (Fig 1). The initial phase of campaign has focused on two primary message themes in television advertisements: (1) novel, visceral, and age-relevant portrayals of the health consequences of smoking; and (2) the loss of control and independence as a result of smoking, which are presented to youth as “the real cost” of smoking.

The media purchase for *The Real Cost* included advertising on national television, online, radio, out-of-home, magazine, and cinema. Overall, the campaign delivered 1,975 mean total television target rating points (TRPs) on national broadcast and cable television from February through June 2014. An additional 1,197 digital TRPs also aired during the time period. Radio advertising aired during this period with an average of 465 planned radio TRPs per market. The media purchase was complemented by additional campaign messages disseminated through social media, mobile gaming, and partnerships with people that are influential among the target audience. The goal of the media strategy for *The Real Cost* was to reach 75% of youth aged 12 to 17 with campaign messages.

## Methods

### Data Source and Sample

**Fig 2:**
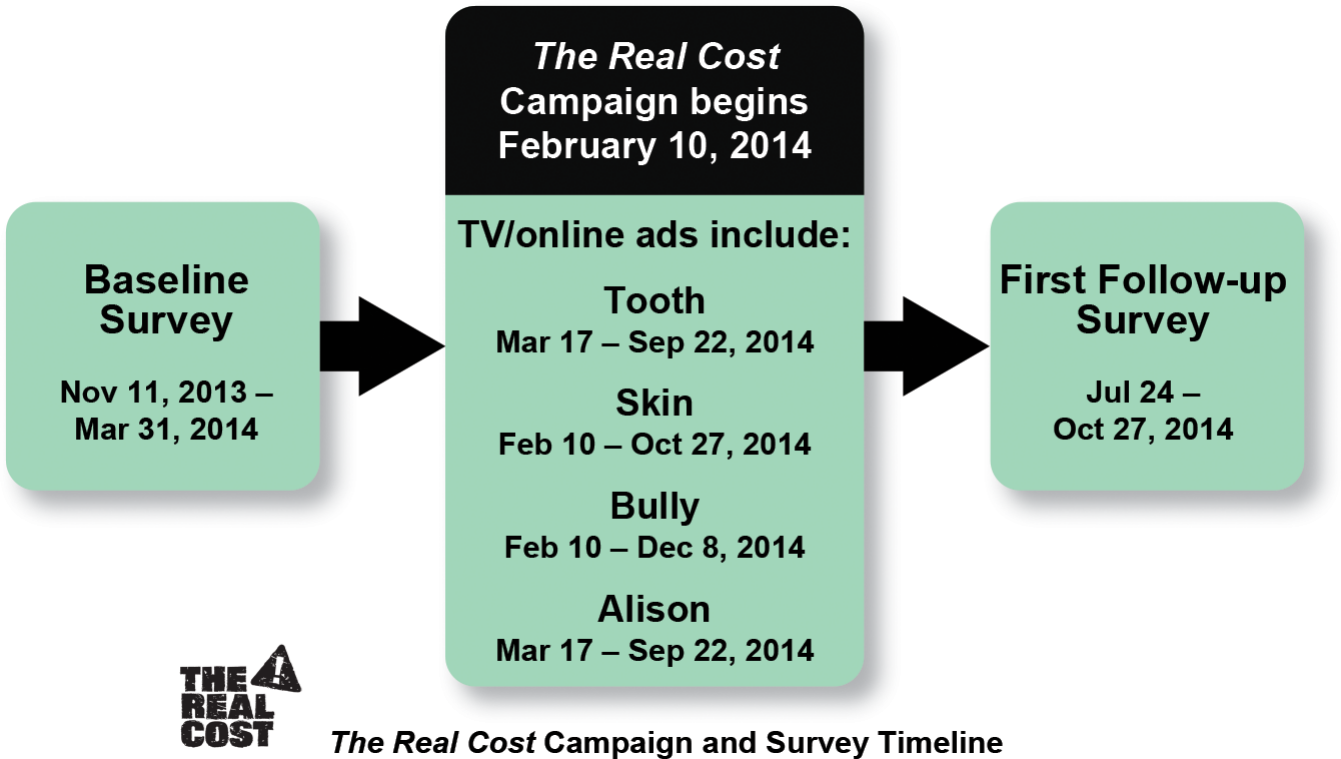
*The Real Cost* Campaign and Survey Timing.

Data are from a national longitudinal in-home and online survey of U.S. youth conducted in collaboration with RTI International as part of *The Real Cost* evaluation. The study sampling design produced a representative sample of youth aged 11 to 16 at baseline (who would age into *The Real Cost* target audience of 12- to 17-year-olds after campaign launch). An address-based sampling frame supplemented with marketing data was used to target households likely to have at least one youth in the target age range. Data collection occurred in 75 media markets. In-person baseline data collection took place from November 11, 2013, through March 31, 2014 (Fig 2). Study data are from the first follow-up data collection, fielded from July 24 through October 27, 2014, which consisted of online or in-person interviews with youth. Interviews were fielded only after parental permission and youth assent were verbally obtained and recorded by the field interviewer. The study and consent procedures were approved by the Institutional Review Boards at both the Food and Drug Administration (FDA) and RTI International as well as by the Office of Management and Budget (OMB). The final analytic sample size for the first follow-up survey was 5,773 youth, with 65% completing in person and 35% completing online.

At baseline, the unweighted household-level response rate was 47.8%, and the weighted household-level response rate was 43.7% (using the American Association of Public Opinion Research Response Rate #3 formula). At follow-up, the unweighted person-level response rate was 86.8%, and the weighted person-level response rate was 85.6%. To produce reliable estimates of target population parameters, baseline analysis weights that accounted for unequal probabilities of selection at each stage were adjusted for nonresponse at follow-up. Then, weights were calibrated to the Census 2010 population totals of the baseline target population with post-stratification for gender and race/ethnicity.

### Measures

#### Outcome Variables

*Brand Awareness*: The survey item used to measure brand awareness was, “In the past 3 months, have you seen or heard the following slogan or theme?” with a response option of “yes” or “no.”

*Awareness of Individual Advertisements*: To assess awareness of the four television advertisements for *The Real Cost*, which aired before the first follow-up interviews, respondents viewed each ad once via video streams during the survey. After viewing each ad, respondents immediately completed questions assessing their frequency of exposure to the ad in the past 3 months; response options were “never,” “rarely,” “sometimes,” “often,” or “very often.” Overall ad awareness was measured as a response of “rarely” or greater to any of the four ads. These procedures are a validated method of measuring individual ad exposure and have been shown to capture encoded exposure more precisely than tests that rely on memory recall alone [21].

*Perceived Effectiveness (PE) Scale*: Youth reactions and receptivity to each ad were assessed immediately after each ad was viewed (ads may be viewed at https://www.youtube.com/user/USFoodandDrugAdmin/videos). PE was measured with a multi-item scale similar to those used in previous studies (e.g., Davis et al. [16,22]). After viewing each ad in the survey, respondents were asked whether they agreed or disagreed with the following statements: (1) this ad was worth remembering, (2) this ad grabbed my attention, (3) this ad was powerful, (4) this ad was informative, (5) this ad was meaningful, and (6) this ad was convincing. Each of these questions was answered on a 5-point Likert-type response scale from 1 (*strongly disagree*) to 5 (*strongly agree*).

A scale was created by summing the items and dividing by six to return the scale to its original item metric (i.e., 1 = *strongly disagree* to 5 = *strongly agree*). Consistent with prior published data [16,22], principal factor analysis suggests a strong one-factor solution for each scale. Factor analysis for the PE scale yielded eigenvalues from 4.1 to 4.3 and factor loadings ranging from 0.73 to 0.90 across the six items in the scale and across all ads for which the scale was measured. All other factor eigenvalues were small and/or negative, and scale reliability was high across all ads (average Cronbach’s alpha = 0.93).

#### Demographic and Smoking-Related Variables

Demographic measures associated with tobacco use were examined, including age, gender, race/ethnicity, and preferred language (English or Spanish) among Hispanic respondents. Smoking-related variables include personal use, tobacco use in the household, and smoking susceptibility measures [12,13]. Among never smokers (not even one or two puffs), three items assessed future intentions to smoke: (1) “Do you think that you will try a cigarette soon?”; (2) “Do you think you will smoke a cigarette anytime during the next year?”; and (3) “If one of your best friends offered you a cigarette, would you smoke it?” with response options “definitely yes,” “probably yes,” “probably not,” or “definitely not.” Youth who responded “definitely not” to all of these items were categorized as non-susceptible nonsmokers, whereas all others (those giving any response other than “definitely not” to any item) were defined as susceptible nonsmokers. Youth who reported smoking fewer than 100 cigarettes in their lifetime were defined as experimenters. Youth who reported smoking more than 100 cigarettes in their lifetime were defined as current or former smokers. Missing responses for the demographic and smoking-related variables were less than 1.1%.

### Analyses

Descriptive statistics summarize data on *The Real Cost* campaign awareness and receptivity to advertisements. T-tests were used to examine demographic characteristics associated with sample attrition and to examine differences in awareness and PE among demographic groups. Demographic and smoking-related variables were used to stratify the data across population subgroups. At follow-up, current or former smokers were excluded from smoking status subgroup analyses due to small sample size (n = 76). All analyses were estimated using Stata statistical software 13.0.

## Results

### Sample Characteristics

The first follow-up sample consisted of 5,761 youth. Respondents in the unweighted sample were evenly distributed across ages 12 to 16, with fewer youth aged 11 and 17 (Table 1). The sample was roughly equivalent by gender; it was 51.7% white, non-Hispanic; 29.0% Hispanic; 9.0% black, non-Hispanic; 3.3% Asian American or Pacific Islander; and 7.1% other or multiracial. Among respondents who reported their ethnicity as Hispanic, Latino/a, or of Spanish origin, a sizable proportion reported being English-only speakers (30.4%). Approximately 30% of the sample constituted the target audience for *The Real Cost*: susceptible nonsmokers (17.5%); and experimenters (12.8%). Almost one-third of respondents (30.8%) reported living in the same household as a tobacco user. An analysis of sample attrition from baseline to follow-up found that the samples were similar across age, gender, and household tobacco use categories. The unweighted follow-up sample contained slightly more white respondents, fewer black respondents, fewer smokers, and more non-susceptible youth than the baseline sample; all differences were very small (e.g., mean difference across comparisons was 0.1%) and were accounted for in the first follow-up survey weighting procedures.

**Table 1:** Characteristics of First Follow-up Study Sample.

Characteristic	N	Unweighted %	Weighted %
Age			
11–13	2,199	38.2%	41.7%
14–15	1,989	34.5%	33.0%
16–17	1,573	27.3%	25.3%
Gender			
Female	3,008	52.2%	49.3%
Male	2,753	47.8%	50.7%
Race/Ethnicity			
White, non-Hispanic	2,981	51.7%	56.0%
Black, non-Hispanic	517	9.0%	12.4%
Hispanic	1,670	29.0%	22.6%
Asian or Pacific Islander	187	3.3%	4.6%
Other or multi-racial	406	7.1%	4.5%
Household Tobacco Use			
Yes	1,759	30.8%	35.9%
No	3,951	69.2%	64.1%
Smoking Status			
Non-susceptible nonsmoker	3,925	68.4%	67.5%
Susceptible nonsmoker	1,005	17.5%	15.7%
Experimenter	733	12.8%	13.7%
Former smoker	19	0.3%	0.6%
Smoker	57	1.0%	2.4%
Language^a^			
Spanish only	16	0.8%	1.2%
English and Spanish	1,391	68.8%	60.7%
English only	614	30.4%	38.1%

^a^ This question was asked of Hispanic or Latino participants or those responding with “Prefer not to answer” to the question “Are you Hispanic, Latino/a, or of Spanish origin?” (n = 2,033).

#### Awareness of *The Real Cost* Brand and Advertisements

**Fig 3:**
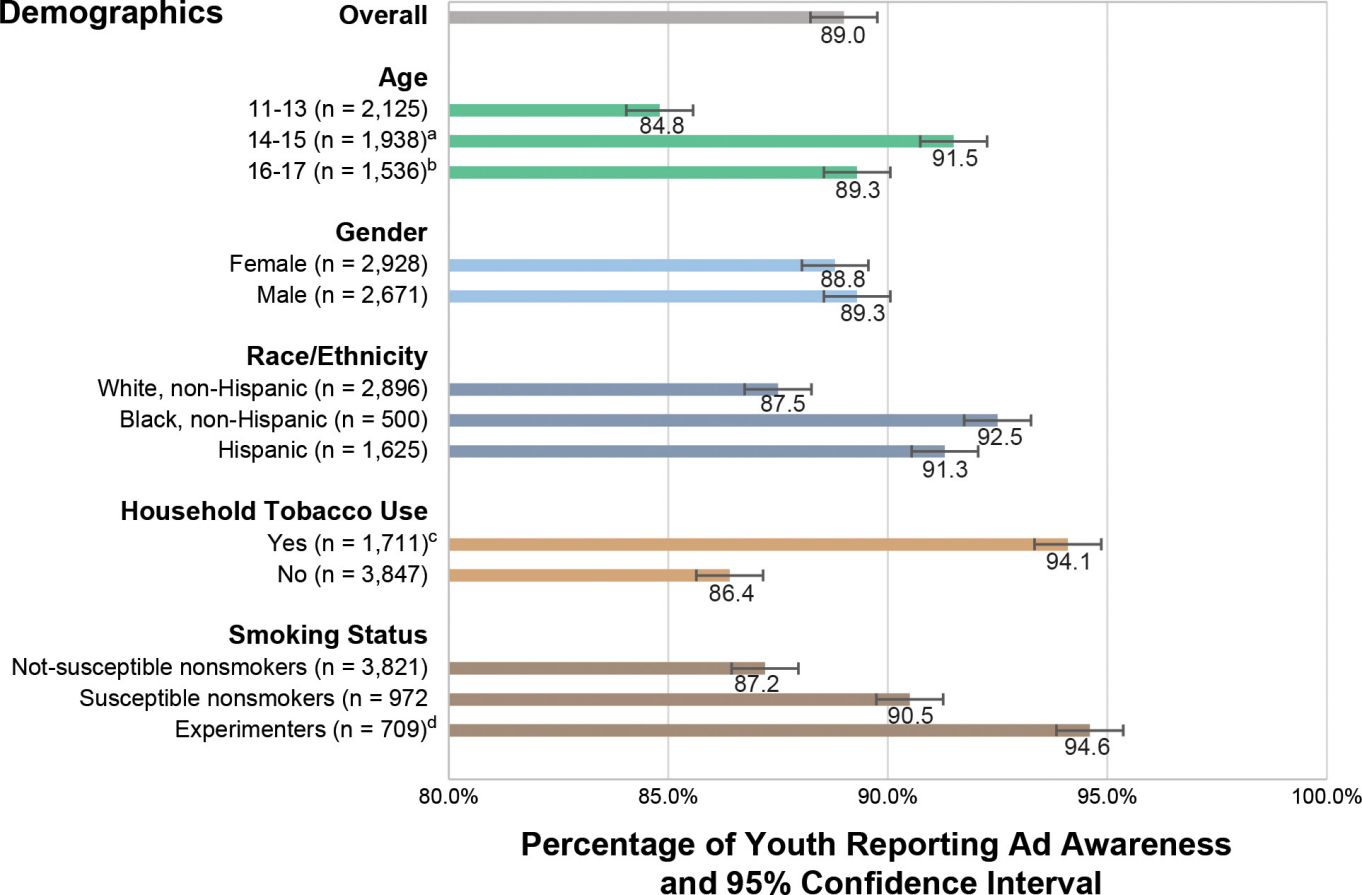
Percentage of Youth Reporting *The Real Cost* Ad Awareness, Overall and by Demographic and Smoking-related Variables. ^a^ 14–15 is significantly higher than 11–13 (*p* < 0.01). ^b^ 16–17 is significantly higher than 11–13 (*p* < 0.01). ^c^ Youth with household tobacco use is significantly higher than youth without (*p* < 0.001). ^d^ Experimenters are significantly higher than non-susceptible nonsmokers (*p* < 0.001).

Awareness of *The Real Cost* brand was 49.9% among youth. Awareness of one or more *The Real Cost* advertisements was 89.0% (Fig 3). Awareness of any ad was higher among youth aged 14 to 17 than among youth aged 11 to 13 (*p* < 0.01). Awareness did not differ significantly by gender or race/ethnicity. Ad awareness was higher among youth experimenters (94.6%) than among non-susceptible nonsmokers (87.2%) (*p* < 0.001). Ad awareness was not statistically different between youth experimenters and susceptible nonsmokers (90.5%). Awareness was significantly higher among youth living with a smoker (94.1%) than youth living in smoke-free households (86.4%) (*p* < 0.001).

Of the four television advertisements, youth were most likely to recall “Skin” (79.6%), followed by “Bully” (76.2%), “Tooth” (76.1%), and “Alison” (67.2%). For “Alison,” “Skin,” and “Tooth,” awareness was higher among youth experimenters (range 74.7% to 84.8%) than among non-susceptible nonsmokers (64.1% to 77.5%, all *p* < 0.05). Youth aged 14 to 17 reported higher awareness than youth aged 11 to 13 for these same ads (70.4% to 83.2% compared with 62.5% to 74.4%, all *p* < 0.05). Awareness of “Skin” was significantly higher among Hispanic youth (85.6%) than among white youth (76.5%) (*p* < 0.01). Awareness of “Alison” and “Skin” was higher among black youth than among white youth (75.6% and 84.8% compared with 64.9% and 76.5%, respectively, both *p* < 0.05).

#### Perceived Effectiveness

**Fig 4:**
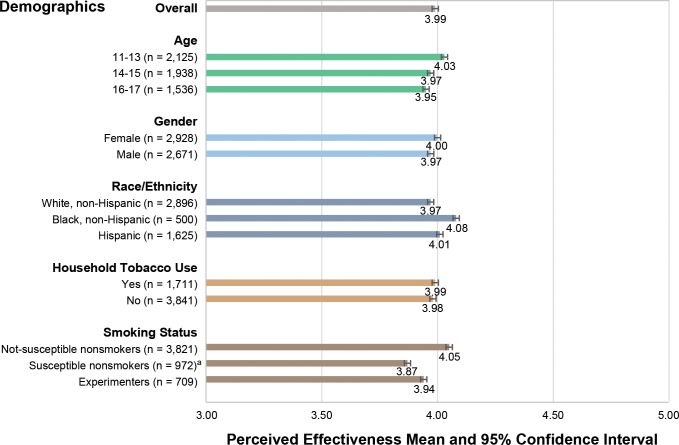
Perceived Effectiveness of The Real Cost Advertisements, Overall and by Demographic and Smoking-related Variables. Note: PE is an average score across the four advertisements on a scale from 1 (lowest) to 5 (highest). ^a^ Susceptible nonsmokers is significantly higher than non-susceptible nonsmokers (*p* < 0.05).

The majority of youth perceived *The Real Cost* advertisements as effective, reporting high levels of agreement with items such as the ad “grabbed my attention” and was “powerful,” “worth remembering,” and “convincing.” Fig 4 displays the mean PE scores for *The Real Cost* ads overall and by key subgroups. Overall, youth reported a mean PE score of 3.99, with individual ad PE scores ranging from 3.91 to 4.07. Mean PE scores did not differ significantly by age, gender, race, or household tobacco use. PE scores were higher among non-susceptible nonsmokers (4.05) than among susceptible nonsmokers (3.87) (*p* < 0.05); experimenters did not differ significantly from the other two groups. PE scores were highest for *The Real Cost* ad “Tooth” (4.08), followed by “Skin” (4.07), “Bully” (3.99), and “Alison” (3.91) (data not shown).

## Discussion

Evidence from this study indicates that the initial phase of *The Real Cost* campaign has exceeded CDC recommendations for achieving population-level awareness levels that are sufficient to potentially produce campaign effects in the future [8]. Half of all youth (49.9%) were aware of *The Real Cost* brand. The vast majority of youth aged 12 to 17 nationwide (89.0%) were aware of at least one television advertisement 6 to 8 months after campaign launch. High levels of awareness were attained for the campaign’s two targeted audiences: susceptible nonsmokers (90.5%) and experimenters (94.6%). A larger proportion of youth experimenters were aware of campaign advertising compared with youth non-susceptible nonsmokers (87.2%). Campaign awareness was also higher among older youth (aged 14 to 17) and youth who lived with a smoker than it was among youth aged 11 to 13 and those who lived in a nonsmoking household. These findings underscore the campaign’s reach among its primary audience of youth at risk for smoking, as research shows that the odds of becoming an established smoker increase with age throughout adolescence, and that living with a smoker is a risk factor for smoking [12,23]. Campaign awareness did not differ significantly by gender or race/ethnicity, suggesting that the campaign reached a diverse population of youth at-risk for tobacco use nationwide. Overall, findings suggest that the campaign is currently generating sufficient exposure within the target audience to ultimately produce behavior change, if other campaign elements, such as messaging, function as expected.

Most youth consider *The Real Cost* advertising to be effective, based on assessments of PE (i.e., the degree to which youth agreed that ads were powerful, convincing, informative, meaningful, worth remembering, and attention grabbing). Because the PE scale ranges from 1 (*strongly disagree*) to 5 (*strongly agree*), the overall mean PE ad score of 3.99 indicates general agreement of effectiveness among study youth. *The Real Cost* advertising was considered more effective among non-susceptible nonsmokers (4.05) than among susceptible nonsmokers (3.87). The ratings of experimenters fell in between (3.94) the other two groups and were not significantly different from either. The PE scores of all three groups are consistent with general agreement about the effectiveness of the advertisements. Consistent with previous studies of PE (Davis et al., 2015, unpublished work) [16], both groups reported positive ad appraisals, with black youth giving *The Real Cost* advertising a higher PE score (4.15) than white youth (3.98). PE scores did not differ significantly by age, gender, or household smoking status. These findings suggest that the campaign was well received overall and within the intended audience.

PE is an important proximal indicator of actual advertising effectiveness. Overall, youth’s positive reactions to *The Real Cost* advertisements are equal to or exceed reactions to previous campaign ads for youth prevention. Data from Florida youth indicate that PE scores across 12 youth tobacco prevention advertisements that had previously aired in the United States ranged from 3.40 to 4.20, with a mean of 3.78 (Duke, 2013, unpublished work). In comparison, the average PE score for four *The Real Cost* advertisements in the study is 3.99, while scores for all subpopulation groups ranged from 3.87 to 4.08. Although there are no youth-focused studies establishing a minimum PE score required for later campaign effectiveness two recent studies of adult cessation advertisements indicate PE is positively associated with subsequent behavioral change. A longitudinal study by Duke et al. [24] found that tobacco cessation advertisements with PE levels ranging from 3.67 to 4.03 subsequently increased adult smokers’ negative feelings about smoking, outcome expectations about the benefits of quitting, and quit intentions. A second study of PE was conducted on the national adult cessation public education campaign *Tips From Former Smokers*, which has been shown to produce cognitive and behavioral effects [25]. The study found that higher PE was predictive of increased quit attempts in a longitudinal sample of adult smokers, with average PE scores across the 15 ads ranging from 3.37 to 4.14 (Davis et al., 2015, unpublished work).

This study is subject to a number of limitations. First, this study relied on an aided recall method in which youth viewed each advertisement prior to reporting awareness, which may have resulted in some overreporting. However, this method is preferable to other measures, such as “confirmed awareness,” which require verbal descriptions of the ending of an ad after viewing the beginning; in the latter method, discrepancies in awareness may occur due to ad characteristics (e.g., narrative-style or non-sequential). For this reason, the aided awareness measure is now commonly used in media campaign evaluations [25]. Second, the PE measure has not been formally validated as predictive of tobacco-related cognitions or behaviors among youth in the published literature, and further evaluation using longitudinal studies is required to determine the influence of PE on youth cognitions and behavioral outcomes. Third, although follow-up data are weighted to account for its effects, sample attrition may limit these data.

Evaluations of previous campaigns have found that high levels of ad awareness and PE were associated with positive changes in campaign-targeted beliefs and attitudes as well as behavior change [4,26–29]). The current study has found similar high levels of ad awareness and PE, suggesting that this campaign has the potential to be effective in reducing smoking among at-risk youth. Initial findings are encouraging and continuing data collections will help determine whether achieving high campaign awareness and PE scores will be associated with future positive youth outcomes for *The Real Cost* campaign.

In conclusion, *The Real Cost* has been widely viewed by youth and appraised as effective, both requisite proximal indicators that have been associated with subsequent changes in health-related beliefs, attitudes, and behavior. Ongoing research using longitudinal panel data will allow for future assessment of the relationship between exposure to the campaign and potential subsequent shifts in youth population-level cognitions and/or behaviors. Currently, these data demonstrate that *The Real* Cost has attained high levels of ad awareness which is a critical first step in achieving positive changes in tobacco-related attitudes and behaviors. These data can also be used to inform ongoing message and media strategies for *The Real Cost* and other U.S. youth tobacco prevention campaigns.

## Supporting Information

S1 File The file includes variables that were used to conduct the analysis reported in this study. (XLSX)

## Abbreviations

CDC: Centers for Disease Control and Prevention
FDA: Food and Drug Administration
